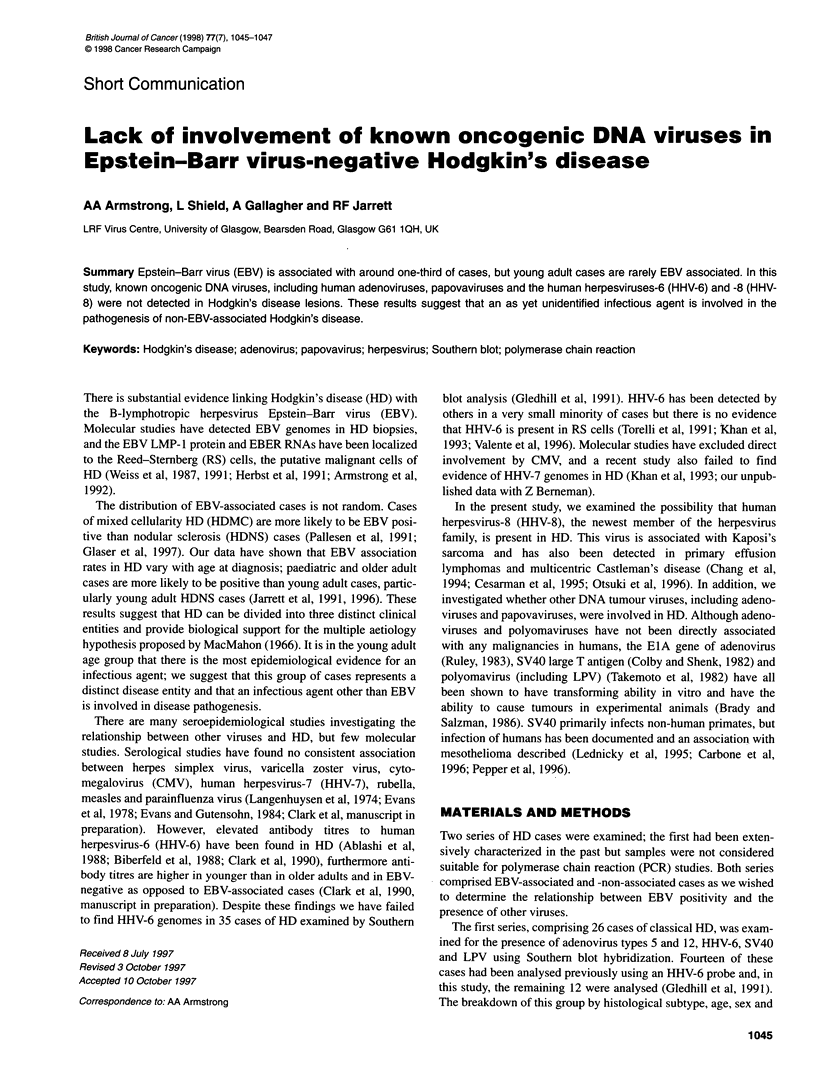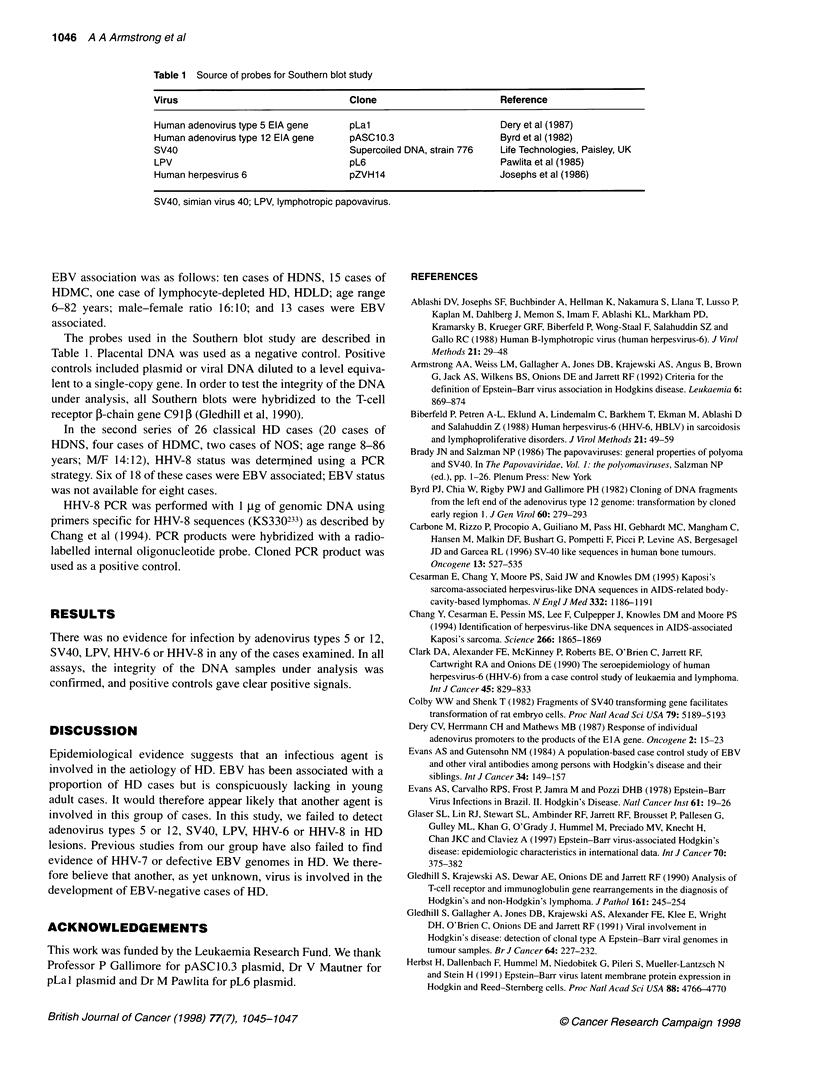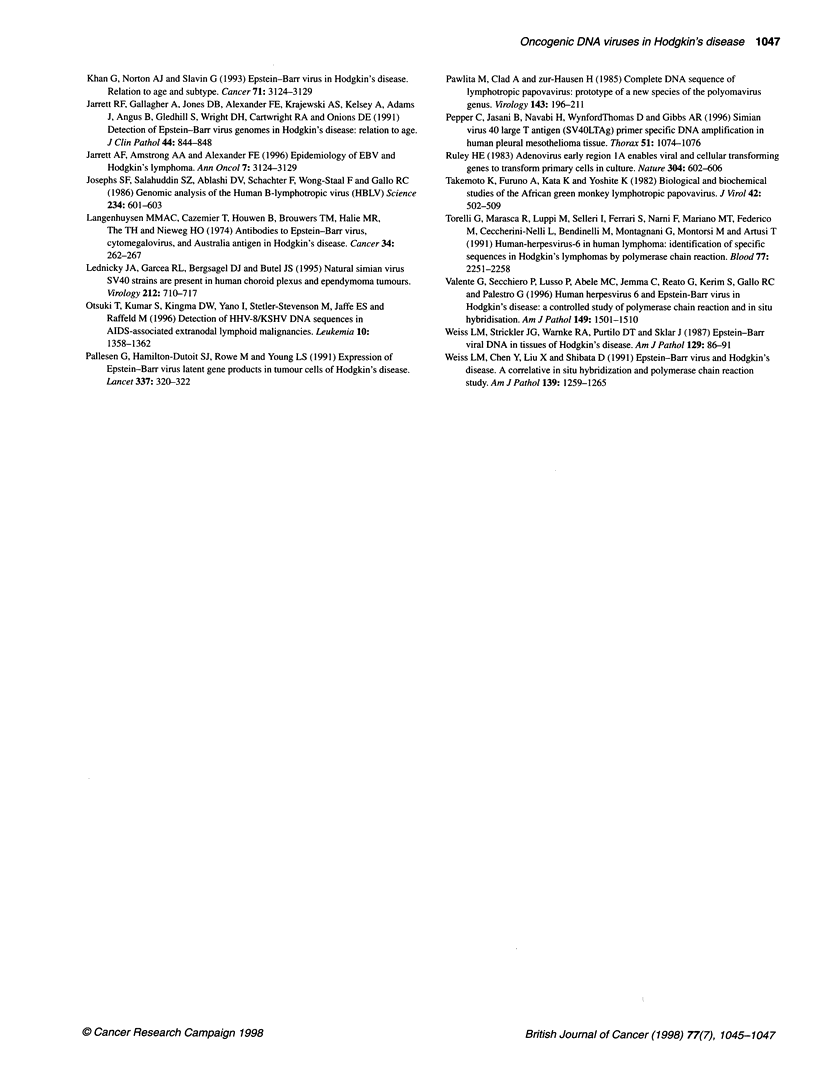# Lack of involvement of known oncogenic DNA viruses in Epstein-Barr virus-negative Hodgkin's disease.

**DOI:** 10.1038/bjc.1998.174

**Published:** 1998-04

**Authors:** A. A. Armstrong, L. Shield, A. Gallagher, R. F. Jarrett

**Affiliations:** LRF Virus Centre, University of Glasgow, UK.

## Abstract

Epstein-Barr virus (EBV) is associated with around one-third of cases, but young adult cases are rarely EBV associated. In this study, known oncogenic DNA viruses, including human adenoviruses, papovaviruses and the human herpesviruses-6 (HHV-6) and -8 (HHV-8) were not detected in Hodgkin's disease lesions. These results suggest that an as yet unidentified infectious agent is involved in the pathogenesis of non-EBV-associated Hodgkin's disease.


British Journal of Cancer (1998) 77(7), 1045–1047
© 1998 Cancer Research Campaign

Short Communication

### Lack of involvement of known oncogenic DNA viruses in Epstein–Barr virus-negative Hodgkin's disease

**AA Armstrong, L Shield, A Gallagher and RF Jarrett**

LRF Virus Centre, University of Glasgow, Bearsden Road, Glasgow G61 1QH, UK

**Summary** Epstein–Barr virus (EBV) is associated with around one-third of cases, but young adult cases are rarely EBV associated. In this study, known oncogenic DNA viruses, including human adenoviruses, papovaviruses and the human herpesviruses-6 (HHV-6) and -8 (HHV-8) were not detected in Hodgkin's disease lesions. These results suggest that an as yet unidentified infectious agent is involved in the pathogenesis of non-EBV-associated Hodgkin's disease.

**Keywords:** Hodgkin's disease; adenovirus; papovavirus; herpesvirus; Southern blot; polymerase chain reaction

There is substantial evidence linking Hodgkin's disease (HD) with the B-lymphotropic herpesvirus Epstein–Barr virus (EBV). Molecular studies have detected EBV genomes in HD biopsies, and the EBV LMP-1 protein and EBER RNAs have been localized to the Reed–Sternberg (RS) cells, the putative malignant cells of HD (Weiss et al, 1987, 1991; Herbst et al, 1991; Armstrong et al, 1992).

The distribution of EBV-associated cases is not random. Cases of mixed cellularity HD (HDMC) are more likely to be EBV positive than nodular sclerosis (HDNS) cases (Pallesen et al, 1991; Glaser et al, 1997). Our data have shown that EBV association rates in HD vary with age at diagnosis; paediatric and older adult cases are more likely to be positive than young adult cases, particularly young adult HDNS cases (Jarrett et al, 1991, 1996). These results suggest that HD can be divided into three distinct clinical entities and provide biological support for the multiple aetiology hypothesis proposed by MacMahon (1966). It is in the young adult age group that there is the most epidemiological evidence for an infectious agent; we suggest that this group of cases represents a distinct disease entity and that an infectious agent other than EBV is involved in disease pathogenesis.

There are many seroepidemiological studies investigating the relationship between other viruses and HD, but few molecular studies. Serological studies have found no consistent association between herpes simplex virus, varicella zoster virus, cytomegalovirus (CMV), human herpesvirus-7 (HHV-7), rubella, measles and parainfluenza virus (Langenhuysen et al, 1974; Evans et al, 1978; Evans and Gutensohn, 1984; Clark et al, manuscript in preparation). However, elevated antibody titres to human herpesvirus-6 (HHV-6) have been found in HD (Ablashi et al, 1988; Biberfeld et al, 1988; Clark et al, 1990), furthermore antibody titres are higher in younger than in older adults and in EBV-negative as opposed to EBV-associated cases (Clark et al, 1990, manuscript in preparation). Despite these findings we have failed to find HHV-6 genomes in 35 cases of HD examined by Southern blot analysis (Gledhill et al, 1991). HHV-6 has been detected by others in a very small minority of cases but there is no evidence that HHV-6 is present in RS cells (Torelli et al, 1991; Khan et al, 1993; Valente et al, 1996). Molecular studies have excluded direct involvement by CMV, and a recent study also failed to find evidence of HHV-7 genomes in HD (Khan et al, 1993; our unpublished data with Z Berneman).

In the present study, we examined the possibility that human herpesvirus-8 (HHV-8), the newest member of the herpesvirus family, is present in HD. This virus is associated with Kaposi's sarcoma and has also been detected in primary effusion lymphomas and multicentric Castleman's disease (Chang et al, 1994; Cesarman et al, 1995; Otsuki et al, 1996). In addition, we investigated whether other DNA tumour viruses, including adenoviruses and papovaviruses, were involved in HD. Although adenoviruses and polyomaviruses have not been directly associated with any malignancies in humans, the E1A gene of adenovirus (Ruley, 1983), SV40 large T antigen (Colby and Shenk, 1982) and polyomavirus (including LPV) (Takemoto et al, 1982) have all been shown to have transforming ability in vitro and have the ability to cause tumours in experimental animals (Brady and Salzman, 1986). SV40 primarily infects non-human primates, but infection of humans has been documented and an association with mesothelioma described (Lednicky et al, 1995; Carbone et al, 1996; Pepper et al, 1996).

### MATERIALS AND METHODS

Two series of HD cases were examined; the first had been extensively characterized in the past but samples were not considered suitable for polymerase chain reaction (PCR) studies. Both series comprised EBV-associated and -non-associated cases as we wished to determine the relationship between EBV positivity and the presence of other viruses.

The first series, comprising 26 cases of classical HD, was examined for the presence of adenovirus types 5 and 12, HHV-6, SV40 and LPV using Southern blot hybridization. Fourteen of these cases had been analysed previously using an HHV-6 probe and, in this study, the remaining 12 were analysed (Gledhill et al, 1991). The breakdown of this group by histological subtype, age, sex and

Received 8 July 1997
Revised 3 October 1997
Accepted 10 October 1997

Correspondence to: AA Armstrong

**Table 1** Source of probes for Southern blot study

| Virus | Clone | Reference |
|---|---|---|
| Human adenovirus type 5 EIA gene | pLa1 | Dery et al (1987) |
| Human adenovirus type 12 EIA gene | pASC10.3 | Byrd et al (1982) |
| SV40 | Supercoiled DNA, strain 776 | Life Technologies, Paisley, UK |
| LPV | pL6 | Pawlita et al (1985) |
| Human herpesvirus 6 | pZVH14 | Josephs et al (1986) |

SV40, simian virus 40; LPV, lymphotropic papovavirus.

EBV association was as follows: ten cases of HDNS, 15 cases of HDMC, one case of lymphocyte-depleted HD, HDLD; age range 6–82 years; male–female ratio 16:10; and 13 cases were EBV associated.

The probes used in the Southern blot study are described in Table 1. Placental DNA was used as a negative control. Positive controls included plasmid or viral DNA diluted to a level equivalent to a single-copy gene. In order to test the integrity of the DNA under analysis, all Southern blots were hybridized to the T-cell receptor β-chain gene C91β (Gledhill et al, 1990).

In the second series of 26 classical HD cases (20 cases of HDNS, four cases of HDMC, two cases of NOS; age range 8–86 years; M/F 14:12), HHV-8 status was determined using a PCR strategy. Six of 18 of these cases were EBV associated; EBV status was not available for eight cases.

HHV-8 PCR was performed with 1 μg of genomic DNA using primers specific for HHV-8 sequences ($KS330^{233}$) as described by Chang et al (1994). PCR products were hybridized with a radiolabelled internal oligonucleotide probe. Cloned PCR product was used as a positive control.

### RESULTS

There was no evidence for infection by adenovirus types 5 or 12, SV40, LPV, HHV-6 or HHV-8 in any of the cases examined. In all assays, the integrity of the DNA samples under analysis was confirmed, and positive controls gave clear positive signals.

### DISCUSSION

Epidemiological evidence suggests that an infectious agent is involved in the aetiology of HD. EBV has been associated with a proportion of HD cases but is conspicuously lacking in young adult cases. It would therefore appear likely that another agent is involved in this group of cases. In this study, we failed to detect adenovirus types 5 or 12, SV40, LPV, HHV-6 or HHV-8 in HD lesions. Previous studies from our group have also failed to find evidence of HHV-7 or defective EBV genomes in HD. We therefore believe that another, as yet unknown, virus is involved in the development of EBV-negative cases of HD.

### ACKNOWLEDGEMENTS

This work was funded by the Leukaemia Research Fund. We thank Professor P Gallimore for pASC10.3 plasmid, Dr V Mautner for pLa1 plasmid and Dr M Pawlita for pL6 plasmid.

### REFERENCES

Ablashi DV, Josephs SF, Buchbinder A, Hellman K, Nakamura S, Llana T, Lusso P, Kaplan M, Dahlberg J, Memon S, Imam F, Ablashi KL, Markham PD, Kramarsky B, Krueger GRF, Biberfeld P, Wong-Staal F, Salahuddin SZ and Gallo RC (1988) Human B-lymphotropic virus (human herpesvirus-6). *J Virol Methods* **21:** 29–48

Armstrong AA, Weiss LM, Gallagher A, Jones DB, Krajewski AS, Angus B, Brown G, Jack AS, Wilkens BS, Onions DE and Jarrett RF (1992) Criteria for the definition of Epstein–Barr virus association in Hodgkins disease. *Leukaemia* **6:** 869–874

Biberfeld P, Petren A-L, Eklund A, Lindemalm C, Barkhem T, Ekman M, Ablashi D and Salahuddin Z (1988) Human herpesvirus-6 (HHV-6, HBLV) in sarcoidosis and lymphoproliferative disorders. *J Virol Methods* **21:** 49–59

Brady JN and Salzman NP (1986) The papovaviruses: general properties of polyoma and SV40. In *The Papovaviridae, Vol. 1: the polyomaviruses*, Salzman NP (ed.), pp. 1–26. Plenum Press: New York

Byrd PJ, Chia W, Rigby PWJ and Gallimore PH (1982) Cloning of DNA fragments from the left end of the adenovirus type 12 genome: transformation by cloned early region 1. *J Gen Virol* **60:** 279–293

Carbone M, Rizzo P, Procopio A, Guiliano M, Pass HI, Gebhardt MC, Mangham C, Hansen M, Malkin DF, Bushart G, Pompetti F, Picci P, Levine AS, Bergesagel JD and Garcea RL (1996) SV-40 like sequences in human bone tumours. *Oncogene* **13:** 527–535

Cesarman E, Chang Y, Moore PS, Said JW and Knowles DM (1995) Kaposi's sarcoma-associated herpesvirus-like DNA sequences in AIDS-related body-cavity-based lymphomas. *N Engl J Med* **332:** 1186–1191

Chang Y, Cesarman E, Pessin MS, Lee F, Culpepper J, Knowles DM and Moore PS (1994) Identification of herpesvirus-like DNA sequences in AIDS-associated Kaposi's sarcoma. *Science* **266:** 1865–1869

Clark DA, Alexander FE, McKinney P, Roberts BE, O'Brien C, Jarrett RF, Cartwright RA and Onions DE (1990) The seroepidemiology of human herpesvirus-6 (HHV-6) from a case control study of leukaemia and lymphoma. *Int J Cancer* **45:** 829–833

Colby WW and Shenk T (1982) Fragments of SV40 transforming gene facilitates transformation of rat embryo cells. *Proc Natl Acad Sci USA* **79:** 5189–5193

Dery CV, Herrmann CH and Mathews MB (1987) Response of individual adenovirus promoters to the products of the E1A gene. *Oncogene* **2:** 15–23

Evans AS and Gutensohn NM (1984) A population-based case control study of EBV and other viral antibodies among persons with Hodgkin's disease and their siblings. *Int J Cancer* **34:** 149–157

Evans AS, Carvalho RPS, Frost P, Jamra M and Pozzi DHB (1978) Epstein–Barr Virus Infections in Brazil. II. Hodgkin's Disease. *Natl Cancer Inst* **61:** 19–26

Glaser SL, Lin RJ, Stewart SL, Ambinder RF, Jarrett RF, Brousset P, Pallesen G, Gulley ML, Khan G, O'Grady J, Hummel M, Preciado MV, Knecht H, Chan JKC and Claviez A (1997) Epstein–Barr virus-associated Hodgkin's disease: epidemiologic characteristics in international data. *Int J Cancer* **70:** 375–382

Gledhill S, Krajewski AS, Dewar AE, Onions DE and Jarrett RF (1990) Analysis of T-cell receptor and immunoglobulin gene rearrangements in the diagnosis of Hodgkin's and non-Hodgkin's lymphoma. *J Pathol* **161:** 245–254

Gledhill S, Gallagher A, Jones DB, Krajewski AS, Alexander FE, Klee E, Wright DH, O'Brien C, Onions DE and Jarrett RF (1991) Viral involvement in Hodgkin's disease: detection of clonal type A Epstein–Barr viral genomes in tumour samples. *Br J Cancer* **64:** 227–232.

Herbst H, Dallenbach F, Hummel M, Niedobitek G, Pileri S, Mueller-Lantzsch N and Stein H (1991) Epstein–Barr virus latent membrane protein expression in Hodgkin and Reed–Sternberg cells. *Proc Natl Acad Sci USA* **88:** 4766–4770

© Cancer Research Campaign 1998

Khan G, Norton AJ and Slavin G (1993) Epstein–Barr virus in Hodgkin's disease. Relation to age and subtype. *Cancer* **71:** 3124–3129

Jarrett RF, Gallagher A, Jones DB, Alexander FE, Krajewski AS, Kelsey A, Adams J, Angus B, Gledhill S, Wright DH, Cartwright RA and Onions DE (1991) Detection of Epstein–Barr virus genomes in Hodgkin's disease: relation to age. *J Clin Pathol* **44:** 844–848

Jarrett AF, Amstrong AA and Alexander FE (1996) Epidemiology of EBV and Hodgkin's lymphoma. *Ann Oncol* **7:** 3124–3129

Josephs SF, Salahuddin SZ, Ablashi DV, Schachter F, Wong-Staal F and Gallo RC (1986) Genomic analysis of the Human B-lymphotropic virus (HBLV) *Science* **234:** 601–603

Langenhuysen MMAC, Cazemier T, Houwen B, Brouwers TM, Halie MR, The TH and Nieweg HO (1974) Antibodies to Epstein–Barr virus, cytomegalovirus, and Australia antigen in Hodgkin's disease. *Cancer* **34:** 262–267

Lednicky JA, Garcea RL, Bergsagel DJ and Butel JS (1995) Natural simian virus SV40 strains are present in human choroid plexus and ependymoma tumours. *Virology* **212:** 710–717

Otsuki T, Kumar S, Kingma DW, Yano I, Stetler-Stevenson M, Jaffe ES and Raffeld M (1996) Detection of HHV-8/KSHV DNA sequences in AIDS-associated extranodal lymphoid malignancies. *Leukemia* **10:** 1358–1362

Pallesen G, Hamilton-Dutoit SJ, Rowe M and Young LS (1991) Expression of Epstein–Barr virus latent gene products in tumour cells of Hodgkin's disease. *Lancet* **337:** 320–322

Pawlita M, Clad A and zur-Hausen H (1985) Complete DNA sequence of lymphotropic papovavirus: prototype of a new species of the polyomavirus genus. *Virology* **143:** 196–211

Pepper C, Jasani B, Navabi H, WynfordThomas D and Gibbs AR (1996) Simian virus 40 large T antigen (SV40LTAg) primer specific DNA amplification in human pleural mesothelioma tissue. *Thorax* **51:** 1074–1076

Ruley HE (1983) Adenovirus early region 1A enables viral and cellular transforming genes to transform primary cells in culture. *Nature* **304:** 602–606

Takemoto K, Furuno A, Kata K and Yoshite K (1982) Biological and biochemical studies of the African green monkey lymphotropic papovavirus. *J Virol* **42:** 502–509

Torelli G, Marasca R, Luppi M, Selleri I, Ferrari S, Narni F, Mariano MT, Federico M, Ceccherini-Nelli L, Bendinelli M, Montagnani G, Montorsi M and Artusi T (1991) Human-herpesvirus-6 in human lymphoma: identification of specific sequences in Hodgkin's lymphomas by polymerase chain reaction. *Blood* **77:** 2251–2258

Valente G, Secchiero P, Lusso P, Abele MC, Jemma C, Reato G, Kerim S, Gallo RC and Palestro G (1996) Human herpesvirus 6 and Epstein-Barr virus in Hodgkin's disease: a controlled study of polymerase chain reaction and in situ hybridisation. *Am J Pathol* **149:** 1501–1510

Weiss LM, Strickler JG, Warnke RA, Purtilo DT and Sklar J (1987) Epstein–Barr viral DNA in tissues of Hodgkin's disease. *Am J Pathol* **129:** 86–91

Weiss LM, Chen Y, Liu X and Shibata D (1991) Epstein–Barr virus and Hodgkin's disease. A correlative in situ hybridization and polymerase chain reaction study. *Am J Pathol* **139:** 1259–1265

© Cancer Research Campaign 1998